# Cancer risk among users of neuroleptic medication: a population-based cohort study

**DOI:** 10.1038/sj.bjc.6603259

**Published:** 2006-08-22

**Authors:** S O Dalton, C Johansen, A H Poulsen, M Nørgaard, H T Sørensen, J K McLaughlin, P B Mortensen, S Friis

**Affiliations:** 1 Institute of Cancer Epidemiology, Danish Cancer Society, 49 Strandboulevarden, Copenhagen, Denmark; 2 Department of Clinical Epidemiology, Aarhus University Hospital, 150 Ole Worms Alle, Aarhus, Denmark; 3 International Epidemiology Institute, Rockville, MD, USA; 4 Department of Medicine, Vanderbilt University Medical Center, Vanderbilt-Ingram Cancer Center, Nashville, TN, USA; 5 National Center for Register-based Research, Aarhus University, Taasingegade 1, Aarhus, Denmark

**Keywords:** neuroleptic medication, population based, cohort study, risk

## Abstract

It has been suggested that neuroleptic medication may decrease cancer risk. We compared cancer risks in a population-based cohort study of 25 264 users (⩾2 prescriptions) of neuroleptic medications in the county of North Jutland, Denmark, during 1989–2002, with that of county residents who did not receive such prescriptions. Statistical analyses were based on age-standardisation and Poisson regression analysis, adjusting for age, calendar period, COPD, liver cirrhosis or alcoholism, use of NSAID, and, for breast cancer, additionally for use of hormone therapy, age at first birth, and number of children. Use of neuroleptic medications was associated with a decreased risk for rectal cancer in both women and men (adjusted IRRs of 0.61 (95% confidence interval, 0.41–0.91) and 0.82 (0.56–1.19), respectively) and for colon cancer in female users (0.78; 0.62–0.98). Some risk reduction was seen for prostate cancer (0.87; 0.69–1.08), but breast cancer risk was close to unity (0.93; 0.74–1.17). Overall, treatment with neuroleptic medications was not related to a reduced risk of cancer, but for cancers of the rectum, colon and prostate there were suggestive decreases in risk.

Neuroleptic medications, in the form of chlorpromazine and other phenothiazine compounds, were introduced into clinical psychiatry in the 1940s and 1950s, and it was proposed early on that these medications might be associated with a reduced cancer risk (Jones, 1985). A recent review concluded that phenothiazines seem to have antiproliferative effects on many tumour cell lines *in vitro* correlating with an antagonistic effect on calmodulin activity, suggesting that calmodulin could be the intracellular target of phenothiazine-induced cell growth inhibition (Hait and Lazo, 1986; Nordenberg *et al*, 1999).

Neuroleptics have been hypothesised to account for the reduced cancer occurrence observed in patients with schizophrenia in a number of studies (Dupont *et al*, 1986; Mortensen, 1989; Gulbinat *et al*, 1992; Mortensen, 1994; Lichtermann *et al*, 2001; Dalton *et al*, 2005). This reduction has been found primarily in men in smoking-related cancers, and in prostate and rectal cancer. Except for a nested case–control study of prostate cancer among Danish schizophrenic in-patients that showed a reduced risk after treatment with chlorpromazine or other high-dose phenothiazines (Mortensen, 1992), the cohort studies of cancer incidence in schizophrenic patients have not included information on use of specific medications.

By contrast, concern has been raised that neuroleptic medication may increase breast cancer risk due to their prolactin-increasing effect (Schyve *et al*, 1978) and this has been reported among women with schizophrenia (Nakane and Ohta, 1986; Gulbinat *et al*, 1992; Lichtermann *et al*, 2001) and a range of psychiatric disorders (Halbreich *et al*, 1996; Wang *et al*, 2002). The few studies of breast cancer risk in patients treated with neuroleptics have mainly found no excess (Overall, 1978; Wagner and Mantel, 1978; Kanhouwa *et al*, 1984; Kelly *et al*, 1999), an exception being a recent cohort study (Wang *et al*, 2002).

We examined risks for cancer overall and for specific sites among adult users of neuroleptic medication in a population-based cohort study of all residents in a Danish county during a 14-year period.

## MATERIALS AND METHODS

The study was conducted within the population of North Jutland, a Danish county with 500 000 inhabitants. The Danish National Health Service provides tax supported health care for all inhabitants, guaranteeing free access to general practitioners and hospitals, and refunds parts of the costs of drugs prescribed by physicians (Olivarius *et al*, 1997). All services are registered by use of the personal identification number, which is assigned to all Danish citizens by the Central Population Register (CPR) and encodes gender and date of birth. Use of the personal identification number secures valid linkage between population-based registries and allows for the establishment of complete cancer and prescription histories for each individual.

### Study population

From the files of the CPR, we identified all 463 232 inhabitants of North Jutland County on 1 January 1989, who were 16–85 years of age during the period 1 January 1989–31 December 2002. We excluded 14 249 individuals with a history of cancer (except nonmelanoma skin cancer) prior to study entry (1989 or age 16 years) by linkage to the nation-wide Danish Cancer Registry, leaving 448 983 persons for follow-up. The Danish Cancer Registry has recorded incident cases of cancer on a nation-wide basis since 1943 and has been shown to have accurate and virtually complete ascertainment of cancer cases (Storm *et al*, 1997).

### Drug exposure data

The North Jutland Prescription Database, established in 1989, holds key information about all prescriptions for refundable drugs dispensed from pharmacies in the county (Gaist *et al*, 1997; Nielsen *et al*, 1997). The data include type of drug prescribed according to the Anatomical Therapeutical Chemical (ATC) classification system, date of dispensing at the pharmacy, and the patient's personal identification number. Using the Prescription Database, we identified 25 264 individuals in the study population who received at least two prescriptions for neuroleptic medication (ATC code N05A) between 1 January 1989 and 31 December 2002 and who were free of cancer at date of second prescription. Persons with only one prescription for neuroleptic medication (*N*=10 877) were not included in this study. ATC code N05A covers the main neuroleptic categories, including phenothiazines, thiozanthenes, butyrophenones, diazepin-oxazepines, benzamides, diphenylbutylpiperidines and indol.

### Psychiatric diagnosis data

The Psychiatric Case Register is a national register that has been computerised since 1 April 1969 and contains data on all admissions to Danish psychiatric in-patient facilities and, since 1995, information from outpatient contacts (Munk-Joergensen and Mortensen, 1997). There is no fee for psychiatric treatment in Denmark, and no private psychiatric facilities exist. The diagnostic system used during the study period was the *International Classification of Diseases,* eighth revision (ICD-8) up to 1993 and 10th revision (ICD-10) thereafter. Using the files of the Psychiatric Case Register, we identified all admissions for psychiatric disorders in the study population from 1969 through 2001. Patients with any hospitalisation or outpatient contact for schizophrenia were identified by ICD-8 code 295 and ICD-10 codes F20 and F25. All other psychiatric in- or outpatients were grouped together.

### Data on other potential confounders

The county Hospital Discharge Registry (HDR) was established in 1977 and contains information on all hospitalisations in the county, including hospital and department number, dates of admission and discharge, and up to 20 diagnoses per hospitalisation and up to six operations per diagnosis (Andersen *et al*, 1999). Diagnoses are coded according to a Danish five-digit version of ICD-8 and ICD-10. We obtained information on all hospitalisations for chronic obstructive pulmonary disorder (COPD) (ICD-8, 490-492; ICD-10, J40-44), liver cirrhosis (ICD-8, 571,573; ICD-10, K70, K72, K74, K76) and alcoholism (ICD-8, 303; ICD-10, F10) between 1978 and 2002, as proxy measures for smoking habits and alcohol consumption that might confound the relationship between use of neuroleptic medications and cancer risk.

From the North Jutland Prescription Database, we further obtained prescription data for medications that potentially could confound the association between neuroleptic medication and cancer risk, including nonsteroidal anti-inflammatory drugs (NSAIDs) (ATC codes; M01A; N02BA01; N02BA51, and B01AC06) and hormone therapy (HT) (ATC groups: G03A-D and G03F).

For a subcohort, we obtained information on age at first childbirth and parity through linkage with the CPR. This information was available for all women born after 1935 and alive in 1968 or later. This restricted the base population in this analysis to 159 942 women and the exposed cohort to 6693 women.

### Follow-up for cancer

Using information from the Cancer Registry and CPR, all individuals in the study population were followed through 31 December 2002 for any first primary diagnosis of cancer from 1 January 1989 or age 16 years, whichever occurred later, and continued until date of cancer diagnosis (except nonmelanoma skin cancer), death, emigration from North Jutland, or 31 December 2002, whichever came first. The person-time of the study subjects was distributed according to use of neuroleptic medication in exposed time (⩾2 prescriptions for neuroleptic medication) and unexposed time (no recorded prescriptions for neuroleptic medication). Person-time between first and second prescription for neuroleptic medication was excluded from the analyses. The exposed time was stratified into number of prescriptions (2–4, 5–9, 10–19, and 20 or more prescriptions).

### Statistical analyses

We computed age- and gender-standardised incidence rates for the exposed group (neuroleptic medication users) by applying direct standardisation in gender-specific 10-year age groups to the age distribution of the entire study population (base population). Age- and gender-standardised incidence rate ratios (IRR) were calculated by dividing cancer incidence rates for neuroleptic users by cancer incidence rates for nonusers of neuroleptic medications (never users). Log-linear Poisson regression analysis, with the logarithm of person-years at risk as an offset variable, was used to compute rate ratios adjusted for calendar period (1989–1994, 1995–1999, 2000–2002), age (16–29, 30–39, 40–49, 50–59, 60–69, 70–84 years), hospitalisations for COPD, liver cirrhosis or alcoholism, and ever use of NSAID. Further, for the subcohort analysis of female breast cancer risk, the rate ratios were adjusted for ever use of HT and for number of children and age at first birth as linear covariates.

Stratified analyses were performed according to sex, number of prescriptions, years of follow-up and psychiatric disorders. Subjects were allowed to change between categories of covariates and exposure variables over time. Within each categorical level all variables were treated as time independent. A test for linear trend was used to evaluate dose response with number of prescriptions and years of follow-up. The statistical analyses were performed in SAS 8.02.

## RESULTS

Characteristics of the cohort of 25 264 neuroleptic users are presented in Table 1. In total, the users of neuroleptic medication accrued 161 478 person-years with a mean follow-up of 6.4 years (range 0–14 years). Compared with female users, more male users were diagnosed with schizophrenia and had a diagnosis of COPD or alcoholism. About a third of the exposed group received 20 or more prescriptions for neuroleptic medication during the study period. The cohort of neuroleptic users overall had a high frequency of psychiatric in- or outpatient contacts, with 6% being hospitalised with schizophrenia and 31% with other psychiatric diagnosis, as well as NSAID use (69%) and morbidity associated with heavy smoking (11%) and alcohol use (9%).

The mean age of the subcohort of 6693 women included in the analysis of breast cancer risk was 43.3 years at entry and mean follow-up was 7.8 years (data not shown). Among these female neuroleptic users, 8.5% had a diagnosis of schizophrenia and a further 33.2% had been hospitalised with another psychiatric disorder (data not shown).

The age- and gender-standardised incidence rate of cancer overall was 626 per 100 000 person-years among neuroleptic users and 543 per 100 000 person-years among never users, yielding an incidence rate ratio (IRR) of 1.15 (95% CI, 1.10–1.21) (Table 2). An increased IRR was observed for lung cancer in both male and female users, whereas a decreased risk was observed for rectal cancer and colon cancer in female users and for rectal cancer in male users (although these latter two risk estimates failed to reach statistical significance). For prostate and breast cancer, age-standardised IRRs were close to unity, however, when restricting the analysis to persons below 67 years of age the IRR for breast cancer went from 1.07 (95% CI, 0.95–1.21) to 0.92 (95% CI, 0.73–1.14) (Table 2). Adjustment for calendar period, diagnoses of COPD, liver cirrhosis, alcoholism, and use of NSAIDs reduced the risk for total cancer and most dramatically for lung cancer in both men and women. The IRR for prostate cancer was also reduced (IRR, 0.87; 95% CI, 0.69–1.08) but the risk estimates for colon or rectal cancer did not change materially; however, the reduced risk for colon cancer in female users reached statistical significance (Table 2). When stratified by type of psychiatric disorder, the rate ratios for prostate cancer and rectal cancer were similarly reduced among patients with schizophrenia, patients with other psychiatric disorders and never hospitalised users of neuroleptic medication (data not shown). For breast cancer, adjustment for parity, age at first birth and use of HT did not change the risk estimate in the subcohort aged 16–66 years. Excluding the first year of follow-up after second prescription for neuroleptic medication yielded similar overall results (data not shown).

There were no significant trends in the adjusted rate ratios by neither number of prescriptions or years of follow-up for cancers of the lung, rectum, colon, prostate or breast, respectively, although the risk estimates were generally increased (lung cancer) or reduced (rectal, colon, prostate and breast cancer) across all strata (data not shown). In persons with more than 10 prescriptions and more than 5 years of follow-up, a low rate ratio was observed for rectal cancer (IRR, 0.47; 95% CI, 0.18–1.26 in men and IRR, 0.33; 95% CI, 0.12–0.88 in women) and for prostate cancer (IRR, 0.66; 95% CI, 0.40–1.10) (data not shown).

## DISCUSSION

We found a reduced risk of rectal cancer in both men and women as well as indications of a reduced risk of colon cancer and prostate cancer in this population-based cohort of neuroleptic users, thus providing some support to the hypothesis of an anticarcinogenic effect of these medications. Reassuringly, we observed no increased risk for breast cancer in female users.

The hypothesis of an anticarcinogenic effect of neuroleptic medications is supported mainly by observations of reduced risks of several cancer types among schizophrenic patients (Dupont *et al*, 1986; Mortensen, 1989; Gulbinat *et al*, 1992; Mortensen, 1994). In this study, we investigated this hypothesis not only among schizophrenic patients but also among other patients receiving antipsychotic treatment. Our cohort study had the advantage of collecting information from population-based databases and registers with prospectively registered and virtually complete data on drug prescriptions, psychiatric and somatic hospital admissions and cancer diagnoses, thus minimising the possibility of selection and information bias. As neuroleptic medications are available only by prescription in Denmark, we are likely to have identified virtually all users of neuroleptic medications in the study population. The fact that a large proportion (37%) of the exposed group had been hospitalised with a psychiatric disorder might have led to some degree of misclassification in the analyses stratified by number of prescriptions, as these patients were probably treated with neuroleptic medication during their hospital admissions, information to which we had no access. Patients with chronic disorders such as psychiatric disorders are likely to be under closer medical supervision and diagnostic activity than the general population possibly resulting in surveillance bias. Conversely, under-ascertainment of cancer among those who are psychotic due to altered perception of physical symptoms and lower degree of adherence to diagnostic activities should also be considered. Although decreased surveillance cannot be ruled out, Danish autopsy studies do not support underdiagnosis of cancers in schizophrenic patients (Mortensen, 1987; Mortensen, 1994).

We observed a decreased risk for rectal cancer in both male and female users of neuroleptic medication. Although there was no clear trend by number of prescriptions or length of follow-up, risk was further reduced among long-term users with more than 10 prescriptions. Studies of patients with schizophrenia in Denmark and Finland have reported reduced risks for rectal cancer but not for colon cancer (Mortensen, 1989; Gulbinat *et al*, 1992; Mortensen, 1994; Lichtermann *et al*, 2001; Dalton *et al*, 2005) whereas we additionally observed a reduced incidence of colon cancer among female users of neuroleptic medication in the present study. Established risk factors for rectal and colon cancer are similar and we would expect exposure to mutual risk factors, such as high intake of alcohol, little exercise and high intake of meat or low intake of vegetables (Potter, 1999), to be more frequent among our cohort of neuroleptic users. Use of NSAIDs was frequent in the present cohort of neuroleptic users, although adjusting for this well established protective factor in colorectal cancer (Thun *et al*, 2002; Baron, 2003) did not alter the risk estimates associated with neuroleptic medication. In schizophrenia, the hypothesis of a lowered host susceptibility to cancer in general has been raised (Catts and Catts, 2000) but the present study indicated that the reduced risk for rectal cancer was not confined to patients with schizophrenia.

Prostate cancer was of *a priori* interest given the reduced risk reported in patients with schizophrenia (Mortensen, 1989; Mortensen, 1994; Lichtermann *et al*, 2001; Dalton *et al*, 2005) and the finding of a reduced risk among men with schizophrenia treated with high-dose phenothiazines (IRR, 0.33; 95% CI, 0.12–0.94) in a small Danish case–control study (Mortensen, 1992). Although in the present study there was no clear trend by number of prescriptions or length of follow-up we did find a 44% lower risk among long-term users with multiple prescriptions. Further, the reduced risk estimates were not confined to patients with schizophrenia but observed in all groups of users of neuroleptic medication, thus supporting an effect of the neuroleptic medication, rather than of schizophrenia.

A rationale for any association between use of neuroleptic medications and breast cancer is that conventional antipsychotic agents inhibit dopamine action at D2 receptors, leading to hyperprolactinemia (Halbreich *et al*, 2003). Epidemiological studies have produced inconsistent findings (Overall, 1978; Schyve, 1978; Wagner & Mantel, 1978; Kanhouwa *et al*, 1984; Nakane *et al*, 1986; Gulbinat *et al*, 1992; Halbreich *et al*, 1996; Lichtermann *et al*, 2001), although a more recent cohort study reported a relative risk of 1.16 (95% CI, 1.07–1.26) for breast cancer associated with use of neuroleptic medication, as well as a positive dose–response relationship (Wang *et al*, 2002). This study included information on a number of factors not included in previous studies, but residual confounding by factors such as reproductive history and family history of breast cancer or incomplete information on socioeconomic status and exogenous hormone use cannot be ruled out (Wang *et al*, 2002). In the present study, we were able to adjust for reproductive factors and use of HT and we found no evidence for an increased breast cancer risk.

Finally, our finding of an increased risk for lung cancer likely reflects the increased smoking among individuals with psychiatric disorders and in treatment with neuroleptic medication. We were able to include certain hospital diagnoses as proxies for heavy smoking and alcohol use; however, given the extent of evidence documenting the increased prevalence of smoking and alcohol use in long-term psychiatric patients (O'Farrell *et al*, 1983; Hughes *et al*, 1986; de Leon *et al*, 1995; Lasser *et al*, 2000), we believe that our results for lung cancer reflect residual confounding by smoking. In support of this, we also observed an increased risk for other smoking related cancers combined, that is cancer of the buccal cavity, oesophagus, pancreas, larynx, urinary bladder, and kidney (Dreyer *et al*, 1997) (adjusted IRR, 1.15; 95% CI, 0.97–1.37) among male users of neuroleptic medications (data not shown).

In conclusion, although no overall decrease in cancer incidence was found, the results of this study provide some support for an anticarcinogenic effect of neuroleptic use for cancers of the rectum, colon and prostate. Reassuringly, we found no increased risk for breast cancer.

## Figures and Tables

**Table 1:** Characteristics at entry of 25 264 adult users of neuroleptic medication in the County of North Jutland, Denmark, 1989–2002

	**Total**		**Male**		**Female**	
**Characteristics**	**No.**	**%**	**No.**	**%**	**No.**	**%**
Total	25 264	100	9708	100	15 556	100
						
Mean age at entry^a^ (years)	58.7		57.3		59.6	
						
Total follow-up (years)	161 478		56 839		104 639	
Mean follow-up (years)	6.4		5.9		6.7	
						
*Age at entry*^a^ *(years)*						
15–29	1672	7	868	9	804	5
30–39	2645	10	1131	12	1514	10
40–49	3950	16	1523	16	2427	16
50–59	4111	16	1476	15	2635	17
60–69	4537	18	1616	17	2921	19
70–85	8349	33	3094	32	5255	34
						
*Year of first prescription*						
1989–94	16 735	66	6036	63	10 699	69
1995–99	5630	22	2411	25	3219	21
2000–02	2899	11	1261	13	1638	11
						
*Number of recorded prescriptions of N05A*						
2–4	8927	35	3582	37	5345	34
5–9	4543	18	1804	19	2739	18
10–19	3726	15	1391	14	2335	15
20+	8068	32	2931	30	5137	32
						
*Psychiatric diagnosis*						
Schizophrenia^b^	1547	6	808	8	739	5
Other^c^	7788	31	2971	31	4817	31
Never	15 929	63	5929	61	10 000	64
						
Diagnosis of COPD^d^	2839	11	1280	13	1559	10
Diagnosis of liver cirrhosis^e^	472	2	223	2	249	2
Diagnosis of alcoholism^f^	1800	7	1118	12	682	4
						
*Use of other drugs* g						
Use of NSAID^h^	17 347	69	6196	64	11 151	72
Use of HT^i^	4786	19	22	<1	4764	31

Neuroleptic medication, ATC NO5A.

a Date of second prescription.

b Schizophrenia, ICD-8: 295; ICD-10: F20, F25.

c Other, hospitalisation with all other psychiatric diagnosis.

d COPD, Chronic obstructive pulmonary disease, ICD-8: 490–492; ICD-10: J40–44.

e Liver cirrhosis, ICD-8: 571,573; ICD-10: K70, K72, K74, K76.

f Alcoholism, ICD-8: 303; ICD-10: F10.

g Two or more prescriptions prior to censoring events.

h Nonsteroidal anti-inflammatory drugs, NSAID, ATC M01A; N02BA01, N02BA51, B01AC06.

i Hormone therapy, HT, ATC G03A, G03C, G03D, and G03F.

**Table 2:** Adjusted incidence rate ratios for selected cancer sites among adult users of neuroleptic medication in the County of North Jutland, Denmark, 1989–2002

	**Neuroleptic use^a^ (*n*=25 264)**		**Never neuroleptic use^b^ (*n*=448 983)**			
	**Number of cases**	**Age-standardised incidence rate^c^**	**Number of cases**	**Age-standardised incidence rate^d^**	**Age-standardised incidence rate ratio (CI)**	**Adjusted incidence rate ratio (CI)^e^**
All sites	1648	626	23 110	543	1.15 (1.10–1.21)	0.99 (0.94–1.04)
Men	622	626	11 531	529	1.18 (1.09–1.28)	1.03 (0.95–1.12)
Women	1026	626	11 579	558	1.12 (1.05–1.19)	0.99 (0.93–1.06)
						
Lung	308	118	3222	76	1.57 (1.40,1.75)	1.24 (1.10–1.40)
Men	127	128	1984	91	1.40 (1.17–1.67)	1.09 (0.91–1.31)
Women	181	109	1238	60	1.81 (1.56–2.10)	1.36 (1.16–1.60)
						
Colon	139	46	2062	49	0.94 (0.79–1.11)	0.88 (0.74–1.05)
Men	56	49	977	45	1.09 (0.82–1.41)	1.10 (0.84–1.44)
Women	83	43	1085	53	0.81 (0.65–1.01)	0.78 (0.62–0.98)
						
Rectum	56	20	1198	28	0.71 (0.54–0.92)	0.70 (0.53–0.92)
Men	29	27	724	33	0.80 (0.54–1.15)	0.82 (0.56–1.19)
Women	27	13	474	23	0.57 (0.38–0.83)	0.61 (0.41–0.91)
						
Breast^f^	258	155	3022	145	1.07 (0.95–1.21)	1.06 (0.93–1.21)
Breast^g^	83	89	1510	97	0.92 (0.73–1.14)	0.93 (0.74–1.17)
						
Prostate^h^	83	119	1595	124^i^	0.96 (0.77–1.19)	0.87 (0.69–1.08)

a Two or more prescriptions for neuroleptic medication redeemed.

b No prescription for neuroleptic medication redeemed.

c Analyses were based on 56 839 person-years of follow-up in men and 104 639 person-years of follow-up in women.

d Person-years of observation: 2 198 464 in men and 2 106 003 in women.

e Adjusted for age, calendar period and diagnosis of COPD, liver cirrhosis, alcoholism and ever use of NSAID.

f Adjusted for age, calendar period and diagnosis of COPD, liver cirrhosis, alcoholism, ever use of NSAID and HT.

g Adjusted for age, calendar period and diagnosis of COPD, liver cirrhosis, alcoholism, ever use of NSAID, age at first child birth, number of children and ever use of HT; This analysis was based on 52 418 women-years of follow-up.

h Restricted to men above 40 years of age, based on 47 603 men-years of follow-up.

i Person-years of observation: 1 287 640.

## References

[bib1] Andersen TF, Madsen M, Jorgensen J, Mellemkjoer L, Olsen JH (1999) The Danish National Hospital Register. A valuable source of data for modern health sciences. Dan Med Bull 46: 263–268 10421985

[bib2] Baron JA (2003) Epidemiology of non-steroidal anti-inflammatory drugs and cancer. Prog Exp Tumor Res 37: 1–24 12795046 10.1159/000071364

[bib3] Catts VS, Catts SV (2000) Apoptosis and schizophrenia: is the tumour suppressor gene, p53, a candidate susceptibility gene? Schizophr Res 41: 405–415 10728718 10.1016/s0920-9964(99)00077-8

[bib4] Dalton SO, Mellemkjær L, Thomassen L, Mortensen PB, Johansen C (2005) Risk for cancer in a cohort of patients hospitalized for schizophrenia in Denmark, 1969–1993. Schizophr Res 75: 315–324 15885523 10.1016/j.schres.2004.11.009

[bib5] de Leon J, Dadvand M, Canuso C, White AO, Stanilla JK, Simpson GM (1995) Schizophrenia and smoking: an epidemiological survey in a state hospital. Am J Psychiatry 152: 453–455 7864277 10.1176/ajp.152.3.453

[bib6] Dreyer L, Winther JF, Pukkala E, Andersen A (1997) Tobacco smoking. APMIS 105: 9–47 9462818

[bib7] Dupont A, Møller Jensen O, Strömgren E, Jablensky A (1986) Incidence of cancer in patients diagnosed as schizophrenic in Denmark. In ten Horn GHMM, Giel R, Gulbinat W, Henderson JH (eds) Psychiatric Case Registries in Public Health. Amsterdam: Elsevier Science Publisherws, pp 229–239

[bib8] Gaist D, Sorensen HT, Hallas J (1997) The Danish prescription registries. Dan Med Bull 44: 445–448 9377907

[bib9] Gulbinat W, Dupont A, Jablensky A, Jensen OM, Marsella A, Nakane Y, Sartorius N (1992) Cancer incidence of schizoprenic patients. Results of record linkage studies in three countries. Br J Psychiatr 161(Suppl 18): 75–85 1389045

[bib10] Hait WN, Lazo JS (1986) Calmodulin: a potential target for cancer chemotherapeutic agents. J Clin Oncol 4: 994–1012 2423656 10.1200/JCO.1986.4.6.994

[bib11] Halbreich U, Kinon BJ, Gilmore JA, Kahn LS (2003) Elevated prolactin levels in patients with schizophrenia: mechanisms and related adverse effects. Psychoneuroendocrinology 28: Suppl 1: 53–67 12504072 10.1016/s0306-4530(02)00112-9

[bib12] Halbreich U, Shen J, Panaro V (1996) Are chronic psychiatric patients at increased risk for developing breast cancer? Am J Psychiatr 153: 559–560 8599407 10.1176/ajp.153.4.559

[bib13] Hughes JR, Hatsukami DK, Mitchell JE, Dahlgren LA (1986) The prevalence of smoking among psychiatric outpatients. Am J Psychiatr 143: 993–997 3487983 10.1176/ajp.143.8.993

[bib14] Jones GRN (1985) Cancer therapy: phenothiazines in an unexpected role. Tumori 71: 563–569 3909565 10.1177/030089168507100608

[bib15] Kanhouwa S, Gowdy JM, Solomon JD (1984) Phenothiazines and breast cancer. J Natl Med Ass 76: 785–788 6471116 PMC2609832

[bib16] Kelly JP, Rosenberg L, Palmer JR, Rao RS, Strom BL, Stolley PD, Zauber AG, Shapiro S (1999) Risk of breast cancer according to use of antidepressants, phenothiazines, and antihistamines. Am J Epidemiol 150: 861–868 10522657 10.1093/oxfordjournals.aje.a010091

[bib17] Lasser K, Boyd JW, Woolhandler S, Himmelstein DU, McCormick D, Bor DH (2000) Smoking and mental illness. A population-based prevalence study. JAMA 284: 2606–2610 11086367 10.1001/jama.284.20.2606

[bib18] Lichtermann D, Ekelund J, Pukkala E, Tanskanen A, Lönnqvist J (2001) Incidence of cancer among persons with schizophrenia and their relatives. Arch Gen Psychiatr 58: 573–578 11386986 10.1001/archpsyc.58.6.573

[bib19] Mortensen PB (1987) Cancerdiagnostik og autopsifrekvens blandt skziofrene patienter. Ugeskr Laeger 149: 1973–1975 3433420

[bib20] Mortensen PB (1989) The incidence of cancer in schizophrenic patients. J Epidemiol Commun Health 43: 43–47 10.1136/jech.43.1.43 PMC1052789 2592890

[bib21] Mortensen PB (1992) Neuroleptic medication and reduced risk of prostate cancer in schizophrenic patients. Acta Psychiatr Scand 85: 390–393 1351334 10.1111/j.1600-0447.1992.tb10325.x

[bib22] Mortensen PB (1994) The occurence of cancer in first admitted schizophrenic patients. Schizophr Res 12: 185–194 7914430 10.1016/0920-9964(94)90028-0

[bib23] Munk-Joergensen P, Mortensen PB (1997) The Danish Psychiatric Central Register. Dan Med Bull 44: 82–84 9062767

[bib24] Nakane Y, Ohta Y (1986) The example of linkage with a cancer register. In ten Horn GHMM, Giel R, Gulbinat W, Henderson JH (eds) Psychiatric Case Registries in Public Health. Amsterdam: Elsevier, pp 240–245

[bib25] Nielsen GL, Sorensen HT, Zhou W, Steffensen FH, Olsen J (1997) The pharmacoepidemiologic prescription database of North Jutland – a valid tool in pharmacoepidemiological research. Int J Risk Safety Med 10: 203–205 10.3233/JRS-1997-10309 23511375

[bib26] Nordenberg J, Fenig E, Landau M, Weizman R, Weizman A (1999) Effects of psychotropic drugs on cell proliferation and differentiation. Biochem Phamacol 58: 1229–1236 10.1016/s0006-2952(99)00156-2 10487524

[bib27] O'Farrell TJ, Connors GJ, Upper D (1983) Addictive behaviors among hospitalized psychiatric patients. Add Behav 8: 329–333 10.1016/0306-4603(83)90032-1 6610282

[bib28] Olivarius NF, Hollnagel H, Krasnik A, Pedersen PA, Thorsen H (1997) The Danish National Health Service Register. A tool for primary health care research. Dan Med Bull 44: 449–453 9377908

[bib29] Overall JE (1978) Prior psychiatric treatment and the development of breast cancer. Arch Gen Psychiatr 35: 898–899 678041 10.1001/archpsyc.1978.01770310104009

[bib30] Potter JD (1999) Colorectal cancer: molecules and populations. J Natl Cancer Inst 91: 916–932 10359544 10.1093/jnci/91.11.916

[bib31] Schyve PM, Smithline F, Meltzer HY (1978) Neuroleptic-induced prolactin level elevation and breast cancer. An emerging issue. Arch Gen Psychiatr 35: 1291–1301 30426 10.1001/archpsyc.1978.01770350017001

[bib32] Storm HH, Michelsen E, Clemmensen IH, Pihl J (1997) The Danish Cancer Registry: history, content, quality and use. Dan Med Bull 44: 535–539 9408738

[bib33] Thun MJ, Henley SJ, Patrono C (2002) Nonsteroidal anti-inflammatory drugs as anticancer agents: mechanistic, pharmacologic, and clinical issues. J Natl Cancer Inst 94: 252–266 11854387 10.1093/jnci/94.4.252

[bib34] Wagner S, Mantel N (1978) Breast cancer at a psychiatric hospital before and after the introduction of neuroleptic agents. Cancer Res 38: 2703–2708 679174

[bib35] Wang PS, Walker AM, Tsuang MT, Orav EJ, Glynn RJ, Levin R, Avorn J (2002) Dopamine antagonists and the development of breast cancer. Arch Gen Psychiatr 59: 1147–1154 12470131 10.1001/archpsyc.59.12.1147

